# Ciclosporin as upfront therapy in Subcutaneous Panniculitis-like T-cell Lymphoma with aggressive features and in uncomplicated disease: Two case reports and literature review

**DOI:** 10.46989/001c.154639

**Published:** 2026-01-05

**Authors:** Brandon K.J. Tan, Kiat Hoe Ong, Yong Howe Ho, Lip Leong Chong, Christian A. Gallardo

**Affiliations:** 1 Department of Haematology, Tan Tock Seng Hospital, Singapore, Singapore; 2 Lee Kong Chian School of Medicine, Nanyang Technological University, Singapore; 3 Yong Loo Lin School of Medicine, National University of Singapore, Singapore; 4 Department of Laboratory Medicine, Tan Tock Seng Hospital, Singapore; 5 Molecular Diagnostic Laboratory, Tan Tock Seng Hospital, Singapore; 6 Department of Pathology, Tan Tock Seng Hospital, Singapore, Singapore

**Keywords:** Subcutaneous panniculitis-like T-cell lymphoma (SPTCL), Ciclosporin, T-cell lymphoma

## To the Editor,

Subcutaneous panniculitis-like T-cell lymphoma (SPTCL) is a rare subtype of cutaneous T-cell lymphoma, accounting for less than 1% of cases. It generally exhibits an indolent course but can be relapsing, refractory, or even complicated by hemophagocytic lymphohistiocytosis (HLH). It usually manifests as subcutaneous nodules, plaques, or ulcers, predominantly on the limbs and trunk. Histologically, it demonstrates a lobular panniculitis pattern with adipocyte rimming by atypical CD8+ cytotoxic T-lymphocytes.

Corticosteroids are often effective, but durable responses are uncommon with monotherapy.^1^ Chemotherapy and stem cell transplantation (SCT) are generally considered for refractory or HLH-complicated disease.^2^ Ciclosporin, though historically used in relapsed or refractory cases, has emerged as a potential front-line agent. Herein, we present two cases of SPTCL, one with aggressive disseminated disease and HLH features, successfully managed with upfront ciclosporin and corticosteroids, underscoring its efficacy and favourable safety profile.

**Figure 1.**
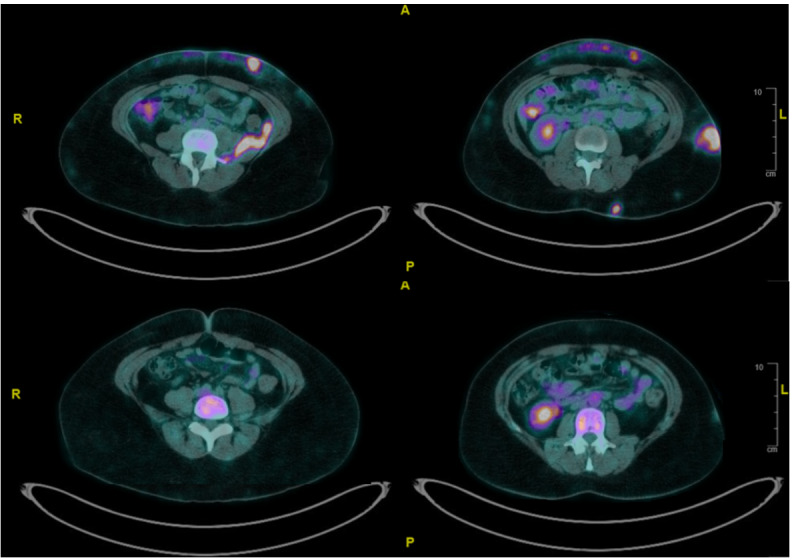
PET scan of abdomen of first case with disseminated SPTCL (top) before treatment: FDG avid sub-cutaneous nodules, lymphadenopathy and retroperitoneal soft tissue thickening. (bottom) 4 months into treatment: Previously noted areas of sub-cutaneous thickening and nodularities have resolved, especially in the anterior and left lateral abdominal walls.

Our first case illustrates a 37-year-old woman with systemic lupus erythematosus and prior class III lupus nephritis. She presented with a 3-month history of painful, indurated subcutaneous nodules on her limbs, thorax, and abdomen. Positron Emission Tomography (PET) scan revealed [18F]Fluorodeoxyglucose (FDG)-avid subcutaneous lesions, generalized lymphadenopathy, and retroperitoneal soft tissue thickening (Figure 1). Biopsies from subcutaneous tissue and iliopsoas muscle demonstrated cytotoxic CD8+ T-cell infiltrates with adipocyte rimming, high Ki-67 index (70–80%), and monoclonal TCRB/TCRG gene rearrangements, confirming SPTCL with disseminated disease.

HLH was a concern due to persistent fever, markedly elevated ferritin and hemophagocytosis on bone marrow examination. Our patient was not keen to receive chemotherapy or SCT. She was initiated on ciclosporin 100 mg BD and high-dose prednisolone. Fevers subsided and inflammatory markers normalized over days. A PET scan at 4 months (Figure 1) demonstrated near-complete resolution of FDG-avid lesions. Prednisolone was tapered, and ciclosporin continued with regular disease surveillance.

At 18 months, she developed disease recurrence with axillary nodules and retroperitoneal FDG-avid lesions. Biopsy confirmed relapse. Ciclosporin troughs were detected to be subtherapeutic (60–80 ug/L) and dose was increased to 125mg BD with high-dose prednisolone initiated. She again achieved remission with no further relapses while prednisolone was tapered. There were no significant adverse effects from ciclosporin use.

Our second case was a 54-year-old woman presenting with a three-month history of non-tender right axillary lumps. PET scan showed localized FDG-avid axillary disease, and biopsy confirmed SPTCL. There were no HLH features. She was treated with ciclosporin 50 mg BD and prednisolone 0.5 mg/kg/day, achieving complete metabolic response by 4 months. She remained in remission on low-dose ciclosporin (25 mg/day), without development of adverse effects.

These cases highlight the effectiveness of ciclosporin-based therapy in SPTCL, including a rare, aggressive case with extracutaneous disease and HLH features. Proposed mechanisms of actions of ciclosporin include inhibition of calcineurin, which is necessary for the activation of the nuclear factor of activated T-cells (NFAT), thereby suppressing the transcription of interleukin-2 (IL-2) and other cytokines critical for T-cell activation and proliferation. Recent findings suggest that ciclosporin may have an immunostimulatory effect by activating the mammalian target of rapamycin complex 1 (mTORC1) pathway in CD8+ T cells leading to potent suppression of T-cell proliferation.^3^

There have not been large validated studies determining optimal first line treatment for SPTCL in view of its rare incidence. We summarized relevant literature of SPTCL treated with ciclosporin-based regimens (Table 2). In a recent cohort study,^4^ these regimens yielded a higher complete response (CR) rate (87%) than chemotherapy (58.3%), though the chemotherapy group had more aggressive baseline features, such as hepatosplenomegaly, suggestive of HLH. Other reports describe durable responses to ciclosporin, particularly in relapsed or HLH-associated cases. However, we observed that treatment outcomes of disseminated disease are likely under-represented by current literature, as very few cases involved extracutaneous dissemination, making our first case a rare and instructive example.

**Table 2.** Summary of relevant literature of SPTCL treated with ciclosporin-based regimens

Author et al., Year	Sample Size	Presence of HLH	Extracutaneous Manifestations	Treatment Regimen	Outcome(s)
Tan et al., 2025	2	Present in 1	Yes (retroperitoneum, extensive lymphadenopathy)	Ciclosporin + corticosteroids	2/2 maintained CR
Tirachotikul et al., 2024	93 (45 ciclosporin, 48 chemotherapy)	Present in some	None specified	Ciclosporin based versus chemotherapy	CR: 87% (CsA) vs. 58.3%; 5-year OS: 98% (CsA)
Yamamoto et al., 2022	1	No	Yes (retroperitoneum)	Ciclosporin + corticosteroids	CR maintained
Girdwichai et al., 2020	4	No	None specified	Ciclosporin + corticosteroids	3/4 maintained CR
Iqbal et al., 2014	1	No	Yes (left breast)	Ciclosporin monotherapy	CR maintained
Go et al., 2012	1	Yes	Yes (muscular involvement)	Ciclosporin based after chemotherapy failure	CR maintained after repeated Ciclosporin treatment
Mizutani et al., 2011	12 (1 new + 11 cases reviewed)	Yes (new case), 9/11 in review	None specified	Ciclosporin + corticosteroids	CR maintained (index); 8/11 CR in review cases
Rojnuckarin et al., 2007	4	No	None specified	Ciclosporin based after chemotherapy failure	3/4 maintained CR
Asati et al., 2016, Sullivan et al., 2020, Kanitthamniyom et al., 2023 amongst other isolated case reports of SPTCL with no extracutaneous manifestations	1	Present in some	None specified	Ciclosporin based +/- corticosteroids and other adjuncts	CR maintained in majority of the case reports

Ciclosporin offers several other advantages over chemotherapy. Adverse effects of ciclosporin such as nephrotoxicity and immunosuppression are relatively manageable. In contrast, anthracycline-based polychemotherapy carry risks of cardiotoxicity, myelosuppression, neurotoxicity, and reproductive toxicity. Fertility preservation is particularly relevant in this context, as SPTCL often affects women of childbearing age.

Additionally, oral administration of ciclosporin decreases the need for frequent hospital visits for intravenous access and associated risks of drug extravasation. Overall, a reduction in healthcare visits also help decrease the risk of nosocomial infections and maintain a better quality of life.

In conclusion, ciclosporin in combination with corticosteroids appears to be a highly effective and well-tolerated treatment for SPTCL, including aggressive disseminated presentations with features of HLH. Both our patients achieved durable remissions, reinforcing its utility as a potential frontline therapy. Given its safety profile, fertility-sparing potential, and ease of administration, ciclosporin should be considered a first-line option, particularly in young and fit patients. Disseminated SPTCL remains under-represented in existing literature, and further clinical studies are warranted to validate ciclosporin-based regimens in this setting.

### Authors’ Contribution

Conceptualization: Brandon K.J. Tan, Christian A. Gallardo;

Data curation: Brandon K.J. Tan, Christian A. Gallardo, Yong Howe Ho;

Formal analysis: Brandon K.J. Tan, Christian A. Gallardo;

Funding acquisition: Brandon K.J. Tan, Christian A. Gallardo;

Investigation: Brandon K.J. Tan, Christian A. Gallardo;

Methodology: Brandon K.J. Tan, Christian A. Gallardo;

Project administration: Brandon K.J. Tan, Christian A. Gallardo;

Resources: Brandon K.J. Tan, Christian A. Gallardo;

Software: Brandon K.J. Tan, Christian A. Gallardo;

Supervision: Brandon K.J. Tan, Christian A. Gallardo, Yong Howe Ho, Lip Leong Chong, Kiat Hoe Ong;

Validation: Brandon K.J. Tan, Christian A. Gallardo;

Visualization: Brandon K.J. Tan, Christian A. Gallardo;

Writing – original draft: Brandon K.J. Tan;

Writing – review & editing: Brandon K.J. Tan, Christian A. Gallardo

### Competing Interest

No competing interests were disclosed.

### Ethical Conduct Approval – Helsinki – IACUC

This study was approved based on appropriate institutional ethical guidelines. Informed consent was obtained from both patients.

### Informed Consent Statement

All authors and institutions have confirmed this manuscript for publication.

## Data Availability

All are available upon reasonable request.
